# Quantification of Gly m 5.0101 in Soybean and Soy Products by Liquid Chromatography-Tandem Mass Spectrometry

**DOI:** 10.3390/molecules24010068

**Published:** 2018-12-25

**Authors:** Hongmin Jia, Tianjiao Zhou, Hong Zhu, Li Shen, Pingli He

**Affiliations:** 1 State Key Laboratory of Animal Nutrition, College of Animal Science and Technology, China Agricultural University, Beijing 100193, China; jiahongmin@126.com (H.J.); ztj_tj_cau0116@outlook.com (T.Z.); 2 Key Laboratory of Urban Agriculture (North China), Ministry of Agriculture, Beijing University of Agriculture, Beijing 102206, China; zhuhong80@pku.edu.cn (H.Z.); shenli@bwu.edu.cn (L.S.); 3 Logistics School, Beijing Wuzi University, Beijing 101149, China

**Keywords:** soybean, Gly m 5.0101, β-conglycinin, protein quantification, liquid chromatography-tandem mass spectrometry

## Abstract

Gly m 5.0101, the alpha subunit of β-conglycinin, is one of the major allergens found in soybeans that has been identified as causing an allergic reaction. Here, we developed a quantification method of Gly m 5.0101 with multiple reaction monitoring using the synthetic peptide ^194^NPFLFGSNR^202^ as the external standard. Firstly, the ground soybean was defatted and extracted with a protein extraction buffer. Then the crude extract was on-filter digested by trypsin and analyzed by liquid chromatography-tandem mass spectrometry. The selected peptide exhibited a detection limit of 0.48 ng/mL and a linear relationship in a concentration range from 1.6 to 500 ng/mL (r^2^ > 0.99). The developed method was successfully applied to quantify the Gly m 5.0101 level in dozens of soybean varieties from different sources and soybean products derived from different processing techniques. The developed method could be used to further analyze β-conglycinin in soybean seeds combined with sodium dodecyl sulfate-polyacrylamide gel electrophoresis (SDS-PAGE) analysis.

## 1. Introduction

*Glycine max*, commonly known as soybean, is a major oilseed crop rich in protein and oil that is often used in food and pharmaceuticals [1]. However, soybean is also listed among the eight most common food allergens for infants and adults in the United States and Europe. Allergic symptoms to soybean include skin rashes, face swellings, a drop in blood pressure, gastrointestinal and respiratory reactions, and in some cases anaphylaxis. Meanwhile, fat-free soybean meal is a significant and cheap source of protein for animal feeds. About 97% of soybean meal is used for animal feed and accounts for approximately 70% of all protein sources used in livestock diets. Similarly, soybean allergy also affects the health of animals especially those at a young age [2]. It has been reported that soybean seeds contain a number of allergic proteins, such as glycinin, β-conglycinin and trypsin inhibitor, which can induce hypersensitivity, damage the structure of the small intestinal, reduce nutrient digestibility, and decrease the production performance of animals [3,4,5]. Among them, β-conglycinin is the main allergen in soybean, which accounts for 10.0% to 12.7% of soybean seed or 24.7% to 45.3% of the total seed proteins. It contains three subunits: α subunit (MW, 57–76 kDa), α’ subunit (MW, 57-83 kDa) and β subunit (MW, 42–53 kDa). The percentage of each subunit varies among genotypes: α from 10.4% to 20.8%, α’ from 8.1% to 20.7% and β from 4.5% to 12.9% of total proteins [6,7]. All three subunits have been purified and tested to have allergenic activity [8,9]. Since the antigenic IgG-binding epitopes (S185-R231) have been identified near the N terminus of the α-subunit [10], the allergenic activity of the α subunit is highest among them [11]. Therefore, the α subunit of β-conglycinin, also known as Gly m 5.0101, has been demonstrated to be a major allergen in soybean.

To prevent allergic reactions caused by soybean, researchers have tried to determine how to eliminate the allergenicity of proteins in soybean and soybean products. Some breeding of seed varieties and feed processing methods (such as physical, chemical, and biological types) have been used to reduce the allergenicity of soybean proteins [2]. Since Gly m 5.0101 is an antigen protein with good thermal stability, the common heat treatment can not eliminate its antigenicity [12]. Recently, new feed processing technologies, such as fermentation or expansion, have been applied to reduce the antigenicity of soybean. For example, soybean meal fermented with Saccharomyces cerevisiae has low immunoreactivity [13]. Lactic acid bacteria can also degrade Gly m 5.0101 into peptides [14].

Due to the differences of soybean varieties and processing technologies, the quality of allergic proteins in soybean and soybean products are significantly different. Currently, there is a need for accurate and sensitive methods to identify and quantify soybean allergens to provide the means for users to compare allergen concentrations among different soybean cultivars and evaluate the efficacy of different processing methods. Initially, traditional sodium dodecyl sulfate-polyacrylamide gel electrophoresis (SDS-PAGE) was used to detect and quantify proteins in soybean [7]. However, since the relative position of the electrophoresed proteins depends on the protein characteristics including the primary structure and the presence of certain side chains or prosthetic groups as well as SDS-PAGE conditions including gel type and concentration, pH, temperature and buffer composition, some proteins do not accurately migrate according to their molecular weight [15]. Meanwhile, some soy proteins with similar molecular weight such as basic subunits of glycinin and trypsin inhibitor may migrate to the same band which is difficult to distinguish. In addition, the sensitivity of SDS-PAGE is so low that the content of trace allergenic proteins in soybean cannot be detected. Thus, this method can be inaccurate and variable, and is only used as a qualitative and semi-quantitative method. Recently, immunoassays, such as IgE-immunoblotting and enzyme-linked immunosorbent assays (ELISA), have been the common approach for identification and quantification of soy allergens. For example, ELISA with polyclonal antibodies have been used to detect α subunit of β-conglycinin [9,11,16]. However, the immunoassays may suffer from other limitations such as antibody availability and high variability, and low sensitivity and specificity, especially among allergen protein families. Since Gly m 5.0101 shares 90.14% and 76.2% homology with the α’ and β subunits of β-conglycinin, respectively, it is difficult to avoid a cross-reactivity with the α’ and β subunits by immunoassays [17]. Therefore, an immunoassay is also only used as a screening and semi-quantitative method.

More recently, liquid chromatography tandem mass spectrometry (LC-MS/MS) has been applied as a powerful method for the discovery and quantitative detection of peptides and proteins in a complex matrix containing various proteins [18,19,20]. The protein quantification technology is commonly performed using stable internal or external standard peptides. Compared with ELISA, this technique obtains a sensitive and accurate quantification of targeted protein without a well-characterized antibody. For example, Houston et al. reported a quantitative proteomic analysis of allergens in 20 commercial soy varieties. Ten soy allergens were identified, and eight allergens were quantified using synthetic isotope-labeled peptides as the internal standards [21]. However, since the selected absolute quantification AQUA peptide “LITLAIPVNKPGR” for the determination of Gly m 5.0101 contained internal trypsin cleavage site, Gly m 5.0101 was shown to have a low abundance and a high variation in the study. Thus, the objective of the present study was to develop LC-MS/MS assay for the quantitative determination of Gly m 5.0101 in soybean samples using multiple reaction monitoring (MRM). The method was evaluated for assay linearity, sensitivity, recovery and accuracy. The presence of Gly m 5.0101 traces in soybean product was clearly discernible through the detection of unique standard peptides. Furthermore, combined with SDS-PAGE analysis, the method was used to determine the content of β-conglycinin according to the percentage of each subunit on PAGE gel. Thus, this study will provide a solid foundation for the evaluation of soybean, soybean products, and soybean processing techniques.

## 2. Results and Discussion

### 2.1. Selection of External Standard Peptide

A sequence of Gly m 5.0101 (UniProtKB P13916) was retrieved from UniProtKB (http://www.uniprot.org/). This sequence was digested in silico and used to generate a list of tryptic peptides using the Peptide Cutter (web-based software, http://web.expasy.org/peptide_cutter/). To select a proper peptide as a quantitative peptide, the selection criteria followed the suggestion by Kamiie et al. [22]. The peptide is unique for the Gly m 5.0101. The length of the peptide is between eight and 10 amino acids without methionine, cysteine or histidine residues. There are no posttranslational modifications and single nucleotide polymorphisms, as well as no continuous sequence of arginine or lysine in the digestion region for the complete digestion by trypsin. Since Gly m 5.0101 shows high similarity with the α’ and β subunits of β-conglycinin (90.14% and 76.2%, respectively), it is difficult to find several unique measurable peptides. Furthermore, the physical and chemical properties of the peptide including the instability index and the grand average of hydropathicity were performed by Protparam (web-based software, http://web.expasy.org/cgi-bin/protparam/protparam). Therefore, according to the criteria, we finally only selected one peptide ^194^NPFLFGSNR^202^ from the list of tryptic peptides. The results showed that the peptide was stable and with good hydrophilicity which was prone to synthesize, resolve and preserve. Meanwhile, when Gly m 5.0101 was cut from SDS-PAGE gels, the high-resolution mass spectrometry (Nano LC-Q-Extractive) identification results showed that the peptide could be detected with a higher signal abundance than that of the other peptides (Supplemental Figure S1). Therefore, we finally selected the 194th to 202th amino acid sequence “NPFLFGSNR” as the unique quantitative detection of the peptide for Gly m 5.0101 (Supplemental Figure S2).

### 2.2. Development of LC-MS/MS Method

**Figure 1:**
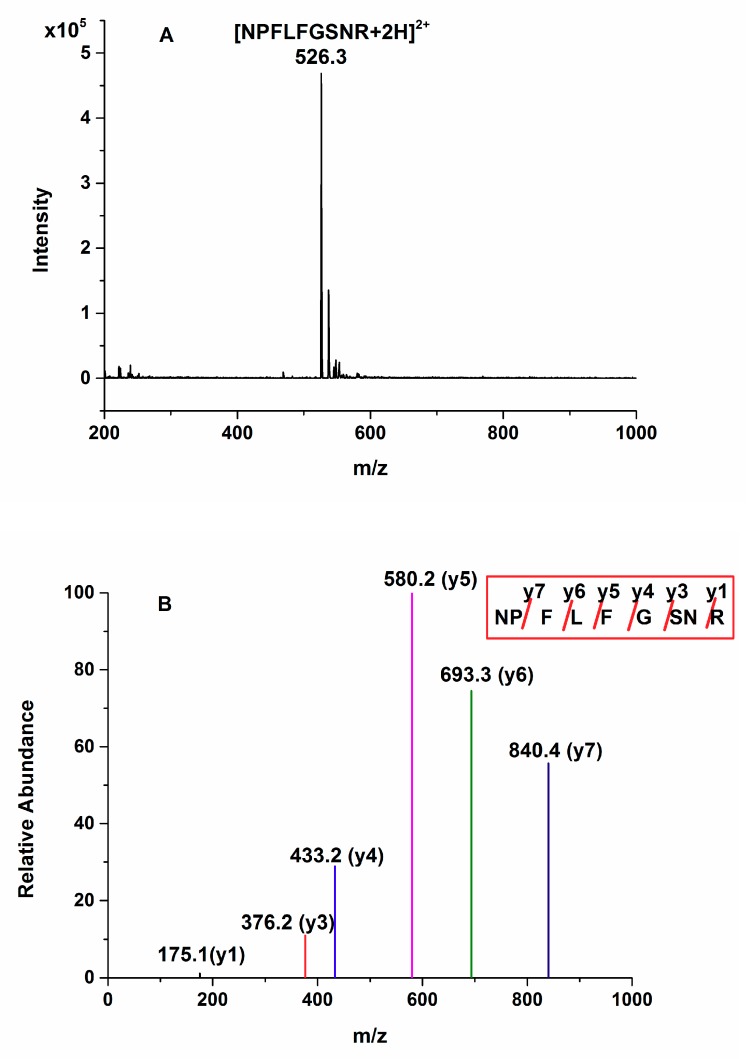
Full-scan (**A**) and product ion (**B**) spectra of the doubly-protonated peptide [NPFLFGSNR + 2H]^2+.^

Since the reversed-phase chromatography column (XBridge^®^ Peptide BEH C18 column, 2.1 × 100 mm, 3.5 μm, Waters, Milford, MA, USA) has been used to separate tryptic peptides and obtain adequate resolution of peptides in a previous report [23], this column was selected to separate the standard peptide with a mobile phase gradient consisting of 0.1% formic acid aqueous solution and acetonitrile (Supplemental Table S1). The 15-min gradient phase program was adequate for the separation of all compounds in the sample. Figure 1A shows the full-scan MS spectrum of NPFLFGSNR in the positive ion mode. The doubly protonated ion of this peptide was observed at m/z 526.3^2+^ with the highest signal intensity. The MS/MS spectrum of this precursor ion is shown in Figure 1B. The intensity of the y5 ion was the highest among the C-terminal y-series ions which is suitable for MRM transition, while the y6 ion had the second highest intensity. Therefore, the ion transitions, m/z 526.3^2+^ to 580.2^+^ and 526.3^2+^ to 693.3^+^ were selected and used for quantification and identification of the peptide, respectively.

**Figure 2:**
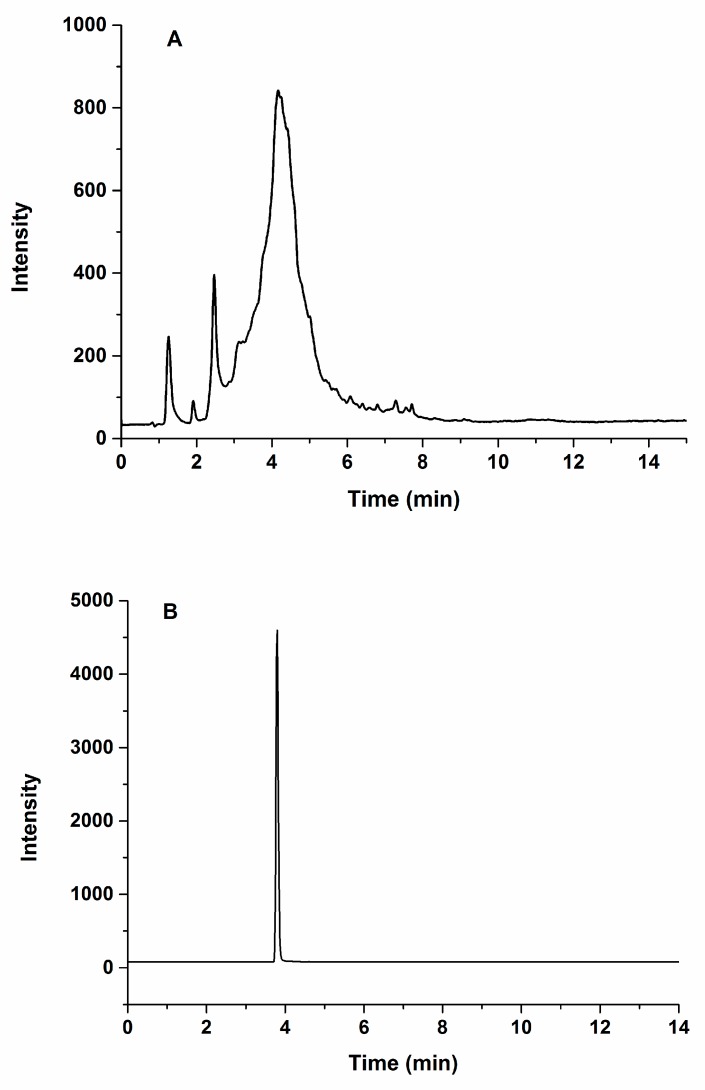
Total ion (**A**) and multiple reaction monitoring (MRM) (**B**) chromatograms of tryptic digestion products from the soybean protein extraction.

Figure 2A,B show the full-scan of total ion and MRM chromatograms of soybean protein extract after tryptic digestion, respectively. The peak corresponding to NPFLFGSNR was clearly and sharply observed at the retention time of 3.89 min on the MRM chromatogram (Figure 2B).

### 2.3. Optimization of Sample Extraction

**Figure 3:**
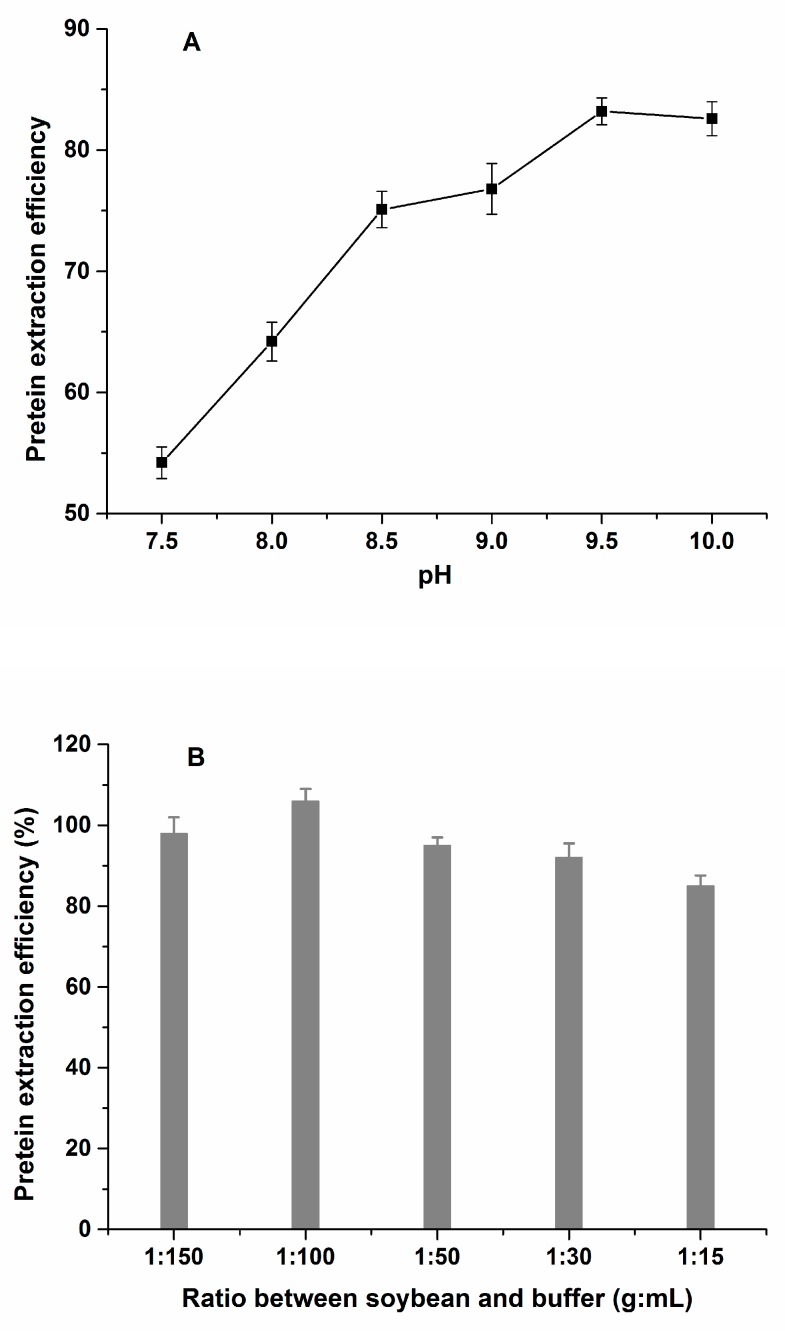

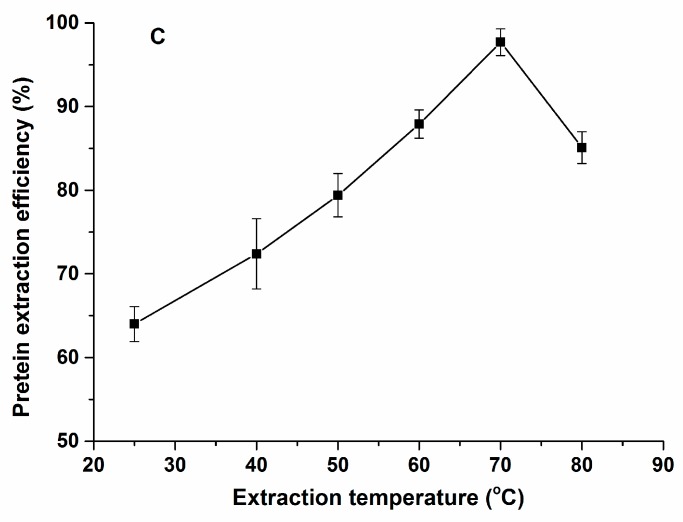
Optimization of sample extraction. Effect of extraction pH (**A**) and ratio between sample and solvent (**B**) on the protein extraction efficiency for the soybean seed; (**C**) effect of extraction temperature on the protein extraction efficiency for the fermented soybean meal.

The development of an efficient procedure for protein extraction is the most important step for the precise quantitative analysis of an analyte. Some soy processing technologies, such as extrusion of soybean meal, need to provide a heat treatment which can denature soy proteins and decrease protein solubility [24]. Therefore, in this study, raw soybean (crude protein content of 41.6%) and fermented soybean meal (crude protein content of 50.2%) were used as test materials to optimize the extract conditions. The conditions that were optimized included the composition of the extraction solvent, pH and salt concentration of the solvent, the ratio of soybean sample to extract solution, and the extraction temperature and time. Many studies have shown that soybean proteins have good solubility in alkaline solution [24,25]. Therefore, Tris-HCl buffer was selected as the extraction buffer, but the optimal salt concentration and pH need further confirmation. After the evaluation of the salt concentrations (25–200 mM) and the pH range (7.5 to 10), the results showed that 50 mM Tris-HCl buffer at pH 9.5 obtained the best solubility of soy proteins (Figure 3A). Here the soybean protein extraction efficiency was calculated by the protein content of soybean sample after extraction divided by the crude protein content before extraction. Urea and sodium dodecyl sulfate (SDS)were used as denaturing agents to dissolve proteins. When adding different concentrations of urea (2, 4, 6, and 8 mol/L) and SDS (0.1%, 0.2%, 0.5% and 1%, w/w) into Tris-HCl buffer, respectively, the results showed that 6 M urea and 0.2% SDS obtained the highest protein content in soybean extract (Figure 3A and Supplemental Figure S3). The ratio between soybean sample and extraction buffer (1g:15 mL, 1g:30 mL, 1g:50 mL, 1g:100 mL, 1g:150 mL and 1g:300 mL) was evaluated. The result showed that 100 mL/g extraction buffer was optimal as this dilution and obtained the highest extraction efficiency of soy proteins (Figure 3B). To increase the protein solubility of soybean products with different processing technologies, heat and shaking treatments were applied. The extraction temperature (25–80 °C) and shaking time (1 h, 2 h and 3 h) were tested. An extraction temperature 70 °C and 2 h shaking time obtained the best solubility of soy proteins in fermented soybean extract (Figure 3C).

### 2.4. Optimization of Sample Digestion

**Figure 4:**
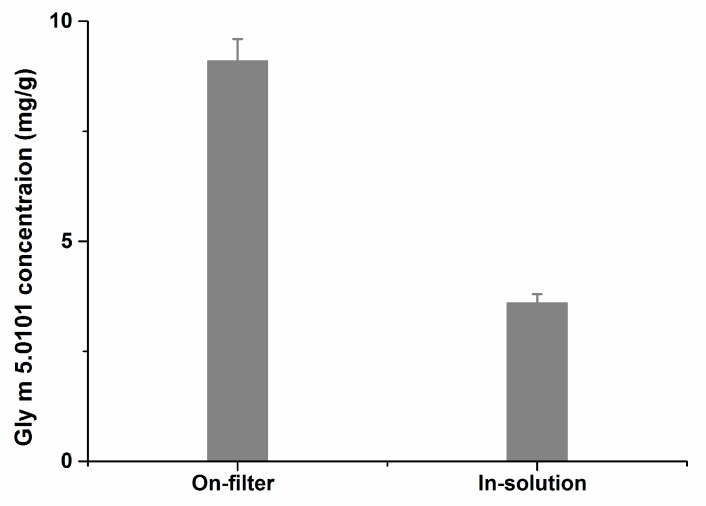
Comparison of different tryptic digestion patterns (on-filter and in-solution) from the soybean protein extraction.

For the determination of target protein by LC-MS/MS, the method of sample digestion has great influence on the quantitative result. Samples from soybean seeds were simultaneously digested by the on-filter or in-solution methods. The results showed that the content of the detected peptide (NPFLFGSNR) derived by on-filter digestion was 2.51 times higher than that of the peptide produced by in-solution digestion (Figure 4). The reason may be that residual dithiothreitol and iodoacetamide in-solution may influence the trypsin activity which lead to insufficient digestion of proteins. However, when using the on-filter method, these residuals may be removed by centrifugation and washing steps before trypsin digestion which eliminated their inhibitory effect on trypsin activity. Thus, on-filter digestion was used as the protein digestion method.

To further identify the tryptic digestion efficiency using the on-filter method, we purified the Gly m 5.0101 from crude β-conglycinin purified by gel. Supplemental Figure S4 shows that the purity of the α subunit (Gly m 5.0101) in lane 3 was approximately 85%. Then, 50 μg of purified α subunit was digested by trypsin on-filter. Results showed that the tryptic digestion efficiency of the method was 85.92%, which demonstrated that it was sufficient to digest the Gly m 5.0101 in 50 μg soybean protein extraction.

### 2.5. Method Validation

The synthetic peptide standard solution at different dilutions showed satisfactory linearity within the concentration range of 1.6–500 ng/mL and correlation coefficients were better than 0.99. The limit of detection (LOD), which was defined as the concentration that could be detected at the signal-to-noise ratio of three, was 0.48 ng/mL for NPFLFGSNR in the soybean extract. The limit of quantitation (LOQ), defined as the concentration that could be detected at the signal-to-noise ratio of ten, was found to be 1.6 ng/mL for NPFLFGSNR in the soybean extract (Supplemental Figure S5). To evaluate the precision and accuracy of the method, the recoveries of peptide spiked in soybean samples were determined using five replicates at different concentrations. The results show that the recoveries of the peptide in spiked soybean samples were between 103.43%–113.13% and intra-day precisions (% CV) were less than 5.91% (Table 1). The inter-day CVs were less than 6.37%. Thus, the developed LC-MS/MS method can be used to accurately determine Gly m 5.0101 in soybean samples.

### 2.6. Quantitation of Gly m 5.0101 in Soybean Seeds and Soybean Products

The developed LC-MS/MS method was able to accurately detect the concentrations of Gly m 5.0101 in soybean samples. The concentrations of Gly m 5.0101 in different soybean seeds ranged from 25.15 mg/g to 41.07 mg/g (Table 2), which was consistent with a previous report [16]. The variation of Gly m 5.0101 may come from regional differences and variation of soybean cultivars. Recently, various processing methods have been developed to reduce antigenic proteins in soybean. In this study, we evaluated the effectiveness of different processing methods in decreasing the content of Gly m 5.0101 in soy products. Three typical soybean products including fermented soybean meal, extruded soybean meal, and extruded full-fat soybean were collected and tested (Table 2). The average concentration of Gly m 5.0101 in fermented soybean meal, extruded soybean meal and extruded full-fat soybean was 9.58 mg/g, 18.35 mg/g, 24.94 mg/g, respectively, which represented a decrease of about 71.35%, 45.14% and 25.44% compared with soybean seeds. Meanwhile, the results also showed that the contents of Gly m 5.0101 have significant differences in different fermented soybean meals, indicating that different fermentation processes had differences in their ability to decrease allergic proteins in soybeans.

### 2.7. Analysis of β-Conglycinin in Soybean Samples

The content of β-conglycinin in each sample could be determined based on the relative quantity of Gly m 5.0101 (the α subunit of β-conglycinin) through the SDS-PAGE method. Supplemental Figure S6 shows that the bands of the three subunits of β-conglycinin were clear and there was no protein with similar molecular weight around the bands. On this basis, we analyzed the proportion of Gly m 5.0101 in β-conglycinin through the gray analysis of total protein gel bands (Table 2). Based on the proportion, the content of β-conglycinin in samples could be calculated by the content of Gly m 5.0101 (Table 2). The results show that the contents of β-conglycinin in fresh soybean seeds ranged from 53.03 to 112.4 mg/g which was consistent with previous reports that β-conglycinin was detected by ELISA methods in soybean seeds ranging from 51.1 to 131.2 mg/g [26,27,28]. The average concentration of β-conglycinin in fermented soybean meal, extruded soybean meal, and extruded full-fat soybean was 42.74 mg/g, 50.70 mg/g and 63.44 mg/g, respectively, which was decreased about 49.17%, 39.71% and 24.56% compared with soybean seeds. Meanwhile, the average concentration of β-conglycinin in fermented soybean meal was 42.74 mg/g, which was also lower than that in extruded soybean meal and extruded full-fat soybean.

Previous research has shown that microbial fermentation could degrade 78.28% of β-conglycinin in soybean meal into small-size peptides or amino acids [29]. Similarly, varying levels of β-conglycinin were observed among the different fermented soybean meals. The changes might be due to differences in microorganisms, basal substrate, fermentation temperature, and other technological parameters during fermentation [30,31]. In addition, since Gly m 5.0101 and β-conglycinin are thermally stable [12,32], the high temperature, high pressure, and shear force applied in the extrusion process have limited ability to destroy all the Gly m 5.0101 and β-conglycinin compared with the fermentation process. Since processing conditions of extruded full-fat soybean including heating temperature, duration of heating and moisture content were not as harsh as that of extruded soybean meal [33,34], there was still an average of 24.94 mg/g Gly m 5.0101 and 63.44 mg/g β-conglycinin detected in extruded full-fat soybean samples.

## 3. Materials and Methods

### 3.1. Materials and Reagents

Ammonium bicarbonate, iodoacetamide, and dithiothreitol were purchased from Sigma Company (St. Louis, MO, USA). Trypsin (Trypsin Gold, Mass Spectrometry Grade) was purchased from Promega (Madison, WI, USA). HPLC grade formic acid and acetonitrile were obtained from Fisher Scientific International (Hampton, NH, USA). BCA™ Protein Assay Kit was obtained from Pierce (Rockford, IL, USA). PageRuler Prestained Protein Ladder 26616 was purchased from Thermo (Rockford, IL, USA). Ultra-pure water with a resistance of 18.2 MΩ cm^−1^ was purified using a Milli-Q system (Millipore, Bedford, VA, USA). All other chemicals were analytic grade. Each liter of Tris-HCl contained 6.057 g Tris and was adjusted to pH 9.5. Nylon membrane filters (0.1 μm) were obtained from Whatman (Maidstone, UK).

The standard peptide NPFLFGSNR was synthesized from GL Biochem. Ltd. (Shanghai, China). The purity of peptide was about 98%–99% according to LC analysis performed by the supplier.

Soybean seeds were provided by the Institute of Crop Science Chinese Academy of Agricultural Sciences (Beijing, China). Soybean products were selected from the Ministry of Agriculture Feed Safety and Bio-availability Evaluation Center (Beijing, China).

### 3.2. Instruments and Apparatus

The LC-MS/MS apparatus used for this study was an Agilent 1200 HPLC system connected with Agilent 6460 QQQ Triple Quad equipped with an electrospray ionization source (Agilent Technologies, Fermont, CA, USA). The identification of Gly m 5.0101 in soybean was performed on a NanoLC-Q-Orbitrap MS system (NanoLC, Waters NanoAcquity HPLC, Milford, MA, USA; MS, Thermo Fisher Q-Exactive, Waltham, MA, USA). An SDS-PAGE electrophoresis slot was purchased from BioRad Laboratories (Herefordshire, England). Gels were digitized with a UMAX Powerlock 2100XL scanner and quantitatively processed with Quantity One software (Shiqun international trade co., LTD, Shanghai, China).

### 3.3. Sample Extraction

The soybean protein extraction was performed as described by our group with some modifications [34]. Briefly, 15 mg soybean sample were ground to a fine powder and extracted by hexane. Defatted soybean flour was subsequently extracted with 1.5 mL protein extraction buffer (50 mM pH 9.5 Tris-HCl containing 6 M urea and 0.2% SDS). The mixture was ultrasonicated at room temperature for 0.5 h, followed by continuous agitation at 1200 rpm for 2 h at 70 °C using a mixer. The suspension was centrifuged at 12000 rpm for 20 min at room temperature. Supernatant was filtered through a 0.2 µm filter and stored at 4 °C until trypsin digestion. The total protein content in the extract was determined by the BCA protein assay reagent kit. Simultaneously, the crude protein content of soybean samples was analyzed using the national standard for crude protein determination.

### 3.4. Trypsin Digestion by on-Filter or in-Solution

The soybean protein extract was digested by trypsin as described by Wisniewski et al. with some modifications [35]. A certain volume of extract (contain 50 μg total protein) was loaded on ultrafiltration filters (10 kDa cutoff, Sartorius, Germany), followed by centrifugation at 14,000*g* for 20 min at room temperature. Then the proteins on-filter were reduced with 50 μL of 10 mM dithiothreitol at 56 °C for 1 h. After centrifugation, the proteins were mixed with 50 μL of 40 mM iodoacetamide and incubated in the dark for 1 h at room temperature to achieve protein alkylation. The mixture was diluted with 300 μL of 50 mM ammonium bicarbonate. The proteins were digested by 40 μL of 25 μg/mL trypsin over night at 37 °C. After incubation, samples were centrifuged at 14,000*g* for 20 min at 4 °C, and mixed with 40 μL 0.2% (*v*/*v*) formic acid, then centrifuged again at 14,000*g* for 20 min at 4 °C.

When samples were digested in solution by trypsin, 50 μg protein sample extracts were mixed with 10 μL of 100 mM dithiothreitol and incubated for 1 h at 56 °C, alkylated with 20 μL 200 mM iodoacetamide in the dark under shaking for 1 h at room temperature, then 20 μL 200 mM dithiothreitol was added to remove the residual iodoacetamide. Samples were digested with 1μg trypsin in 200 mM ammonium bicarbonate at 37 °C overnight, centrifuged at 14,000*g* for 20 min at 4 °C. At last, the digested sample was stored at ‒20 °C until LC-MS/MS analysis.

### 3.5. Analysis of Trypsin Digestion Efficiency

Firstly, crude β-conglycinin was extracted from soybean seeds by the method of Zheng et al. with some modifications [9]. Its three subunits were isolated using SDS-PAGE. The Gly m 5.0101 (α subunit) band were excised from the gel and stained by 0.25 M KCl. The Gly m 5.0101 was extracted by extraction buffer from gel slices at 4 °C over night. After centrifugation at 14,000*g* for 20 min at 4 °C, the supernatant containing the purified Gly m 5.0101 was digested by trypsin according to the on-filter procedure for the analysis of trypsin digestion efficiency.

### 3.6. Nano LC-Q-Extractive Analysis

The identification of Gly m 5.0101 in soybean was performed on a nano HPLC coupled with a Thermo Q-Exactive high resolution mass spectrometer. Sample (7 L) was loaded onto a 100 mm I.D. fused silica capillary trap column filled with 2 cm of C18 stationary phase (Aqua C18, 5 mm, 125 Å Phenomenex, Torrance, CA, USA). The analytical column was a 50 mm I.D. fused silica capillary filled with 10 cm of C18 stationary phase (Aqua C18, 3 mm, 125 Å, Phenomenex). A 90 min gradient was used to elute the peptides (Table 1). Mobile phase A was 0.1% FA in water, B was 0.1% FA in acetonitrile, and the flow rate was 200 nL min^−1^. The nano ESI spray voltage was 2.0 kV and a full mass scan in the range of m/z 300 to 1800 was obtained with a resolution of 70,000 at m/z 200. The 10 most intensive peptide signals from the full scan were selected for the MS/MS scan at a resolution of 17,500 at m/z 200, the maximum injection time was 50 ms, the isolation window was 4 m/z, and the dynamic exclusion time was 20 s.

### 3.7. LC-MS/MS Analysis

For the quantitative analysis of soybean samples, trypsin digestion solution (40 μL) was diluted with 160 μL water containing 0.1% formic acid and centrifuged at 14,000 g for 10 min at 4 °C. The supernatant was transferred into a sample vial. The supernatant (5 μL) was loaded onto XBridge^®^ Peptide BEH C18 column (3.5 μm particle size, 2.1 × 100 mm) at 25 °C. The chromatography separation was accomplished with 0.3 mL/min flow using a water (A)/acetonitrile (B) solution (both phases containing 0.1% (v/v) formic acid) as the mobile phase. A gradient program was used for elution: Starting at 95% A for 0 min, decreasing to 10% A within 7 min, remaining at 10% A for 3 min, returning to the starting conditions immediately and equilibrating the column for 5 min. Subsequently, a multiple reaction monitoring (MRM) method for the peptide with two precursor ion-product ion transitions was developed. The optimized electrospray ionization conditions for peptide were: Gas flow (10 L/min), gas temperature (350 °C), sheath flow (12 L/min), sheath gas temperature (400 °C), and capillary voltage (3500 V). A total ion scan of the mass range m/z 200–1500 with a scan time duration of 0.4 s/scan was set for the full scan of soybean samples. To optimize the MRM intensity derived from NPFLFGSNR, dwell time (150 ms), fragmentor (120 V) and collision energies (20 V) with a scan time duration of 0.4 s/scan for the MRM transitions were derived from repetitive measurements to obtain high signal intensities. The acquisition and analysis of data was performed by Agilent MassHunter Qualitative Analysis software (B.00.10.0).

### 3.8. Validation Procedure

The validity of the LC-MS/MS method, including linearity, sensitivity, as well as recovery and accuracy was evaluated in five replicates. In order to avoid background interference, a fermented soybean meal was chosen as the blank sample to do the method verification experiment, which had been tested without the target peptide segment. The concentration of the standard peptide solution was diluted gradually with a 0.1% formic acid solution to a range of concentrations from 1.6 to 500 ng/mL. The standard peptide diluted with blank soybean extract were analyzed to determine the limit of detection (LOD, S/N = 3) and limit of quantitation (LOQ, S/N = 10) using the LC-MS/MS method. For the recovery analysis, an aliquot of sample after washing by ammonium bicarbonate was mixed with 30, 60, and 120 fmol standard peptide solution, and digested by 1 μg trypsin on ultrafiltration filter over night at 37 °C. This experiment was repeated on three different days.

### 3.9. Analysis of Gly m 5.0101 in Soybean Seeds and Soybean Products

Eleven samples of soybean seeds harvested in 2016 were collected from China with different origin and breed. Seventeen soybean products from three different processing methods, including fermented soybean meal, extruded soybean meal, and extruded full-fat soybeans were prepared in triplicate. The amount of Gly m 5.0101 was detected in each sample using the developed assay by external standard peptide quantitation. The calculation formula was as follows:

m_Gly m 5.0101_ (mg/g) = n_Detected peptide_^a^ (mmol) × 70284.1/m_Sample_ (mg) × 1000

Note: ^a^ The concentration of detected peptide in the sample was determined with a calibration curve obtained from the standard peptide curve.

### 3.10. Analysis of β-Conglycinin in Soybean Seeds and Soybean Products

Firstly, SDS–PAGE was performed to detect the protein profile of soybean samples. Samples (including 50 μg total proteins) were loaded onto 5%–10% polyacrylamide gel and performed in a vertical electrophoresis. The gel was stained with Coomassie Brilliant Blue R-250 (0.05%, *w*/*v*) in methanol-acetic acid-water (25:10:65, *v*/*v*/*v*) and destained in the same solution without dye. PAGE gels were digitized with a UMAX Powerlock 2100XL scanner. The relative quantity of three subunits for β-conglycinin was determined using Quantity One software. According the proportion of Gly m 5.0101 in the whole β-conglycinin, the corresponding contents of β-conglycinin in each soybean sample could be calculated.

## 4. Conclusions

In this study, a sensitive LC-MS/MS analysis using an external standard peptide was successfully developed for the accurate quantification of Gly m 5.0101 in soybean samples. The peak of NPFLFGSNR was clearly detected in the MRM chromatogram and showed a good resolution from other products of tryptic digestion of soybean protein extract. The assay showed an acceptable sensitivity towards NPFLFGSNR peptide with a LOD at 0.48 ng/mL and accuracy with recoveries of 103.43%–113.13%, which was sufficient to detect trace amounts of Gly m 5.0101 in all kinds of soybean seeds and soybean meals. The assay was successfully applied to detect levels of Gly m 5.0101 in soybean seeds and soybean products with different processing techniques. Furthermore, combined with SDS-PAGE analysis, the content of β-conglycinin in soybean samples could also be obtained. Data from soybean seeds showed that regional differences and variation of soybean cultivars has some influence on Gly m 5.0101 and the β-conglycinin content. The extrusion and fermentation could significantly decrease the level of allergic proteins in soybean products. However, the effects have large differences among different processing technologies. Thus, the developed LC-MS/MS method, with its high sensitivity and accuracy, is a very promising technique for the detection and quantification of allergic proteins in soybeans and soybean products.

## Figures and Tables

**Table 1:** Recoveries of the peptide spiked in defatted soybean seed samples (*n* = 5).

Spiked Level (fmol)	Measured Concentration (fmol)	Mean Recovery (%)	Intra-day Precision CV (%)	Inter-day Precision CV (%)
30	31.03	103.43	5.91	6.37
60	65.06	108.44	2.25	2.36
120	135.76	113.13	2.49	2.83

**Table 2:** Concentrations of Gly m 5.0101 and β-conglycinin in actual soybean samples from soybean seeds and soybean products.

No.	Name	Gly m 5.0101 Concentration (mg/g)	The Proportion of Gly m 5.0101 in β-Conglycinin (%)	β-Conglycinin Concentration (mg/g)	No.	Name	Gly m 5.0101 Concentration (mg/g)	The Proportion of Gly m 5.0101 in β-Conglycinin (%)	β-Conglycinin Concentration (mg/g)
1	Southeast spring-summer-autumn soybean seeds	41.00 (0.51) ^a^	37.92 (2.32) ^b^	108.27	12	Fermented soybean meal	0.00	─	0.00
2		41.07 (7.12)	36.63 (3.38)	112.37	13	Fermented soybean meal	0.00	─	─
3		34.60 (3.08)	35.58 (1.62)	97.19	14	Fermented soybean meal	11.19 (4.22)	25.65 (3.78)	43.63
4		28.33 (3.11)	35.31 (3.14)	80.32	15	Fermented soybean meal	21.27 (10.31)	26.53 (2.42)	80.17
5	Huanghuai summer soybean seeds	38.02 (4.52)	36.93 (4.19)	103.15	16	Fermented soybean meal	18.83 (9.72)	26.22 (3.74)	71.82
6		35.71 (3.58)	38.60 (2.84)	92.47	17	Fermented soybean meal	6.15 (9.54)	33.96 (4.63)	18.11
7		25.15 (3.04)	47.41 (1.93)	53.03	18	Extruded soybean meal	10.83 (9.03)	36.36 (2.07)	29.79
8	North spring soybean seeds	30.66 (4.29)	47.02 (4.08)	65.23	19	Extruded soybean meal	17.55 (0.23)	33.04 (3.61)	53.12
9		35.00 (3.17)	46.73 (3.72)	74.95	20	Extruded soybean meal	24.52 (0.73)	36.84 (1.52)	66.56
10		28.41 (2.42)	43.61 (2.53)	65.16	21	Extruded soybean meal	19.83 (2.54)	36.86 (4.28)	53.80
11		30.00 (3.71)	41.28 (4.64)	72.65	22	Extruded soybean meal	16.30 (0.42)	36.06 (2.93)	45.20
					23	Extruded soybean meal	21.06 (4.31)	37.78 (3.48)	55.74
					24	Extruded full-fat soybean	26.19 (1.73)	37.09 (4.68)	70.61
					25	Extruded full-fat soybean	32.64 (3.87)	38.85 (4.88)	84.02
					26	Extruded full-fat soybean	25.86 (3.04)	35.09 (3.82)	73.70
					27	Extruded full-fat soybean	10.24 (2.54)	43.40 (2.68)	23.59
					28	Extruded full-fat soybean	29.75 (3.64)	45.54 (3.41)	65.33

Note: ^a^ Coefficients of variation for detection of Gly m 5.0101 concentration are shown in brackets. ^b^ Coefficients of variation for detection of the proportion of Gly m 5.0101 in β-conglycinin in actual soybean samples are shown in brackets.

## Supplementary Materials

The supplementary materials are available online.
