# Supplementary material for: Quantification of Gly m 5.0101 in Soybean and Soy Products by Liquid Chromatography-Tandem Mass Spectrometry
Source: Molecules. 2018 Dec 25;24(1):68. doi: 10.3390/molecules24010068 (PMC6337133; doi:10.3390/molecules24010068)
Supplement: Supplementary file 1 [file molecules-24-00068-s001.pdf]

Supporting Information

Quantification of Gly m 5.0101 in soybean and soy products by liquid chromatography-tandem mass spectrometry

Tianjiao Zhou[1], Runxian Li[1], Hong Zhu[2], Li Shen[2,3], Pingli He[*,1]

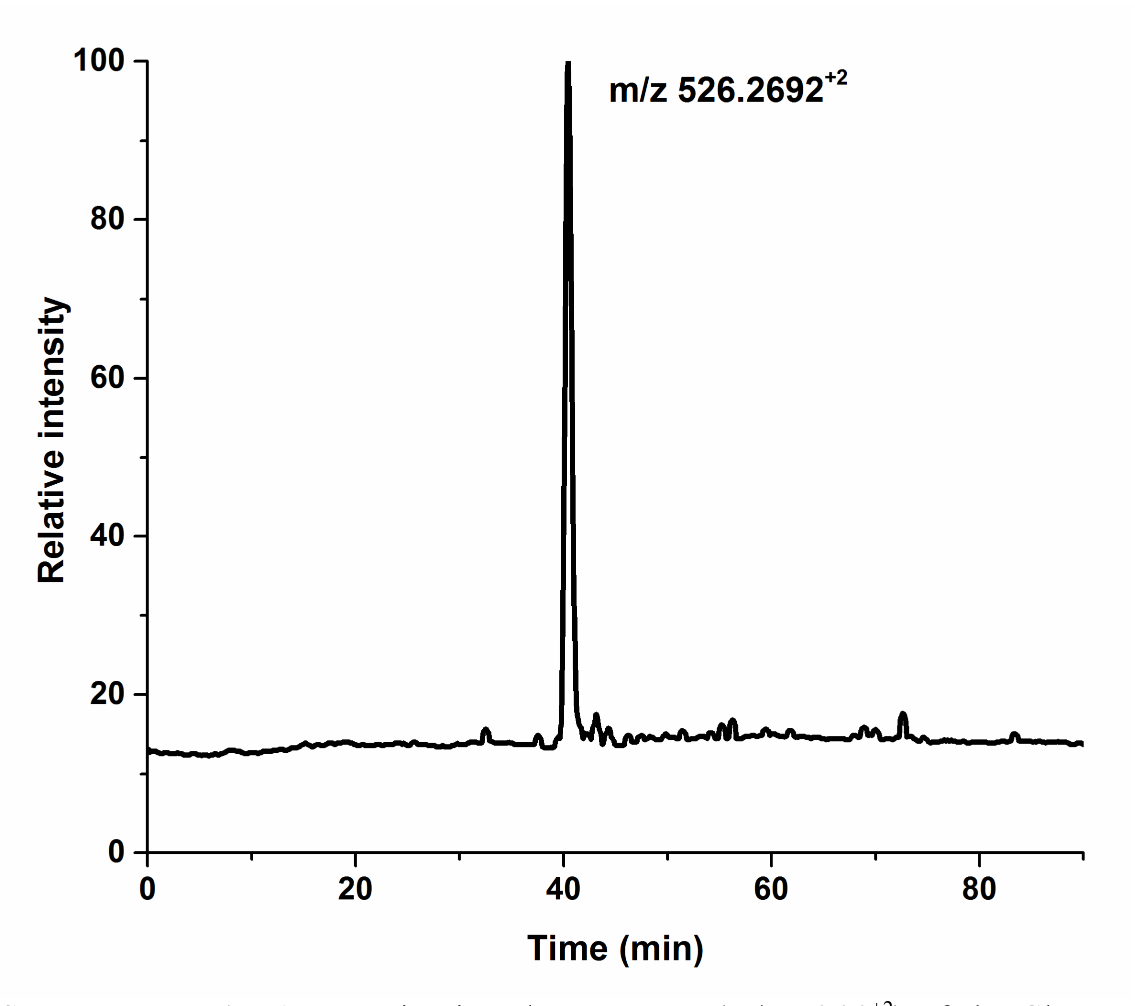

Supplemental Fig. 1 Extractive ion chromatogram (M/Z 526.3^{+2}) of the Gly m 5.0101 from SDS-PAGE gels band using Q-Orbitrap high resolution mass spectrometry.

1	MMRARFPLLL	LGLVFLASVS	VSFGIAYWEK	ENPKHNKCLQ	SCNSERDSYR
51	NQACHARCNL	LKVEKEECEE	GEIPRPRPRP	QHPER**EPQQP**	**GEKEEDEDEQ**
101	**PRPIPFPRPQ**	**PRQEEEHEQR**	**EEQEWPR**KEE	KRGEK**GSEEE**	**DEDEDEEQDE**
151	**RQFPFPRPPH**	**QKEERKQEED**	**EDEEQQRESE**	**ESEDSELRR**H	K**NKNPFLFGS**
201	**NRFETLFKNQ**	**YGR**IRVLQRF	NQR**SPQLQNL**	**R**DYRILEFNS	KPNTLLLPNH
251	ADADYLIVIL	NGTAILSLVN	NDDR**DSYRLQ**	**SGDALRVPSG**	**TTYYVVNPDN**
301	**NENLRLITLA**	**IPVNKPGRFE**	**SFFLSSTEAQ**	**QSYLQGFSRN**	**ILEASYDTKF**
351	**EEINKVLFSR**	**EEGQQQGEQR**	**LQESVIVEIS**	**K**EQIRALSKR	AKSSSR**KTIS**
401	**SEDKPFNLRS**	**RDPIYSNKLG**	**KFFEITPEKN**	**PQLRDLDIFL**	**SIVDMNEGAL**
451	**LLPHFNSKAI**	**VILVINEGDA**	**NIELVGLKEQ**	**QQEQQQEEQP**	**LEVR**KYRAEL
501	SEQDIFVIPA	GYPVVVNATS	NLNFFAIGIN	AENNQR**NFLA**	**GSQDNVISQI**
551	**PSQVQELAFP**	**GSAQAVEK**LL	KNQR**ESYFVD**	**AQPK**KKEEGN	KGRKGPLSSI
601	LRAFY				

Supplemental Fig. 2 The amino acid sequence of Gly m 5.0101 in Mascot database. Italicized/bolded/underlined = selected peptide NPFLFGSNR for AQUA.

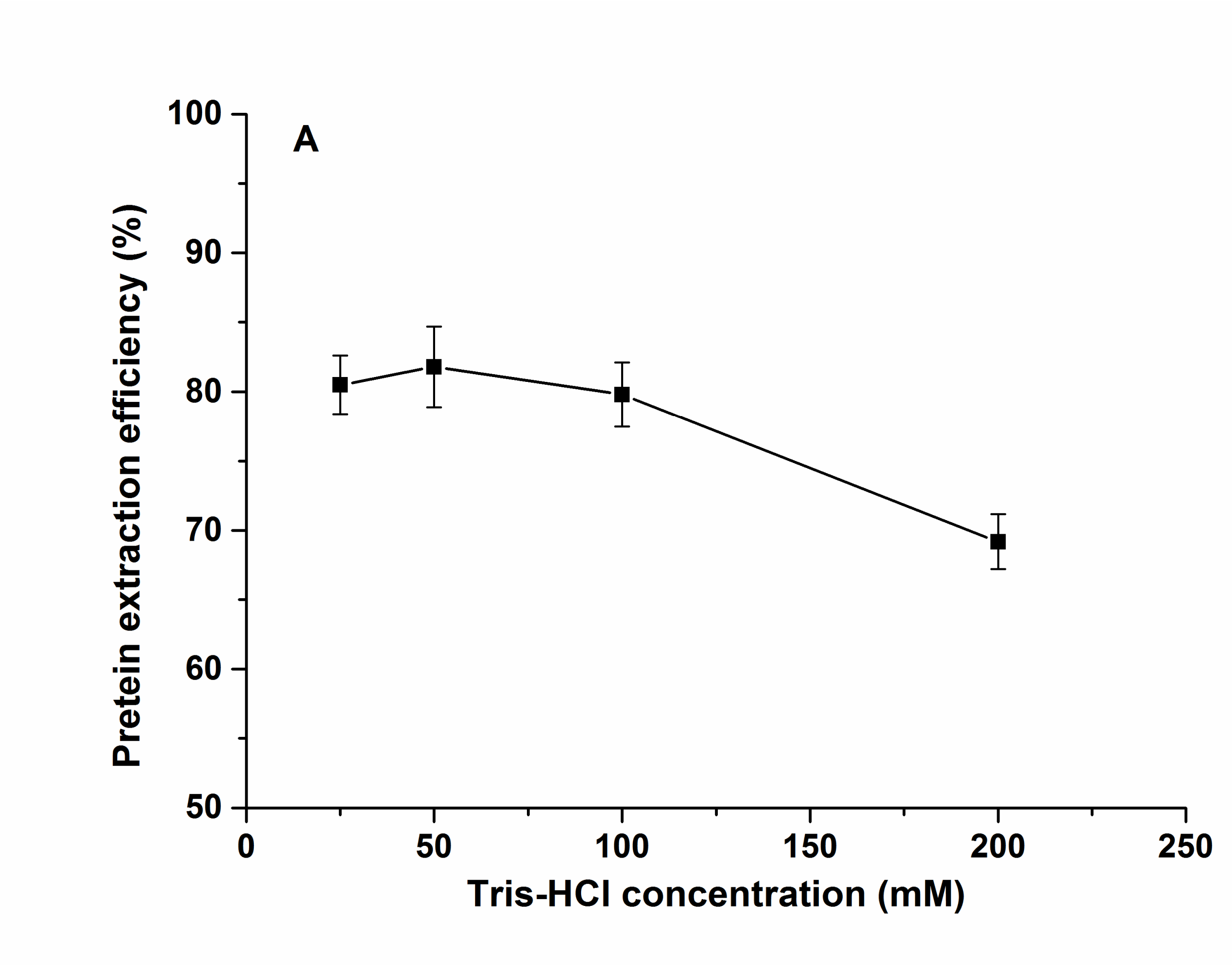
A
Pretein extraction efficiency (%)
Tris-HCl concentration (mM)
100
90
80
70
60
50
0
50
100
150
200
250

1

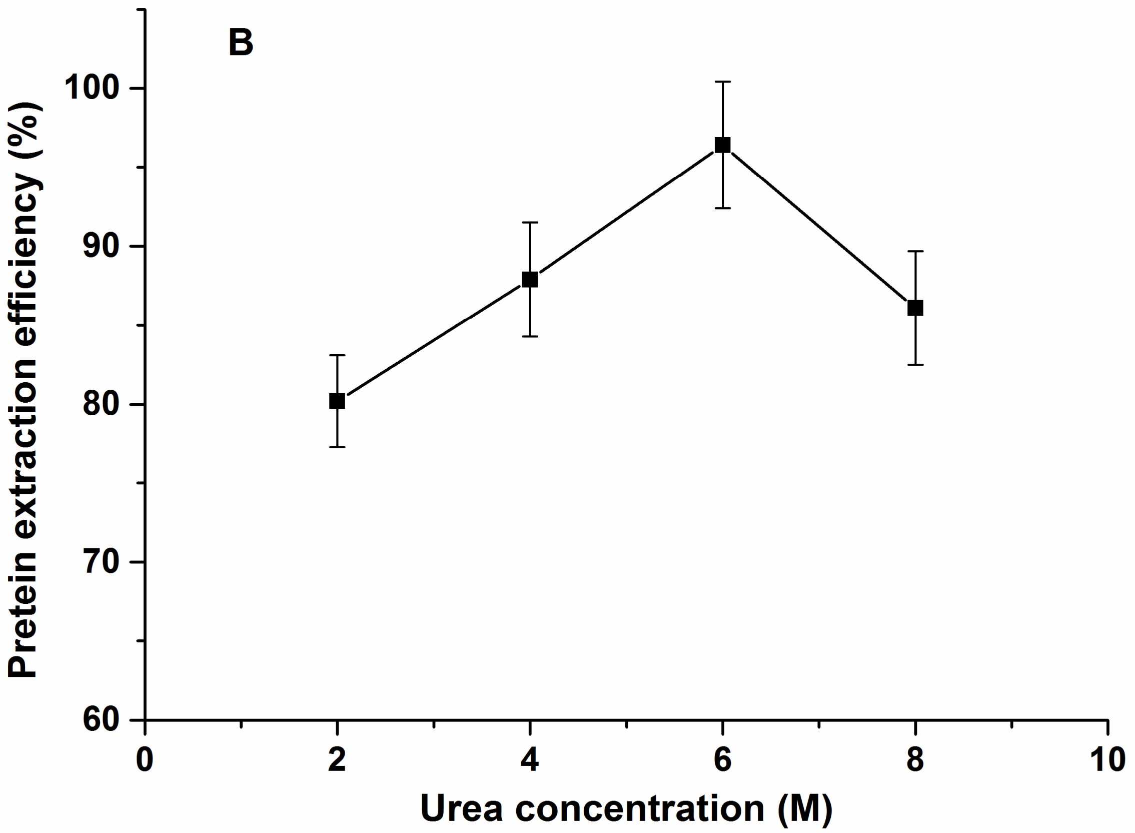
B
Pretein extraction efficiency (%)
60
70
80
90
100
0
2
4
6
8
10
Urea concentration (M)

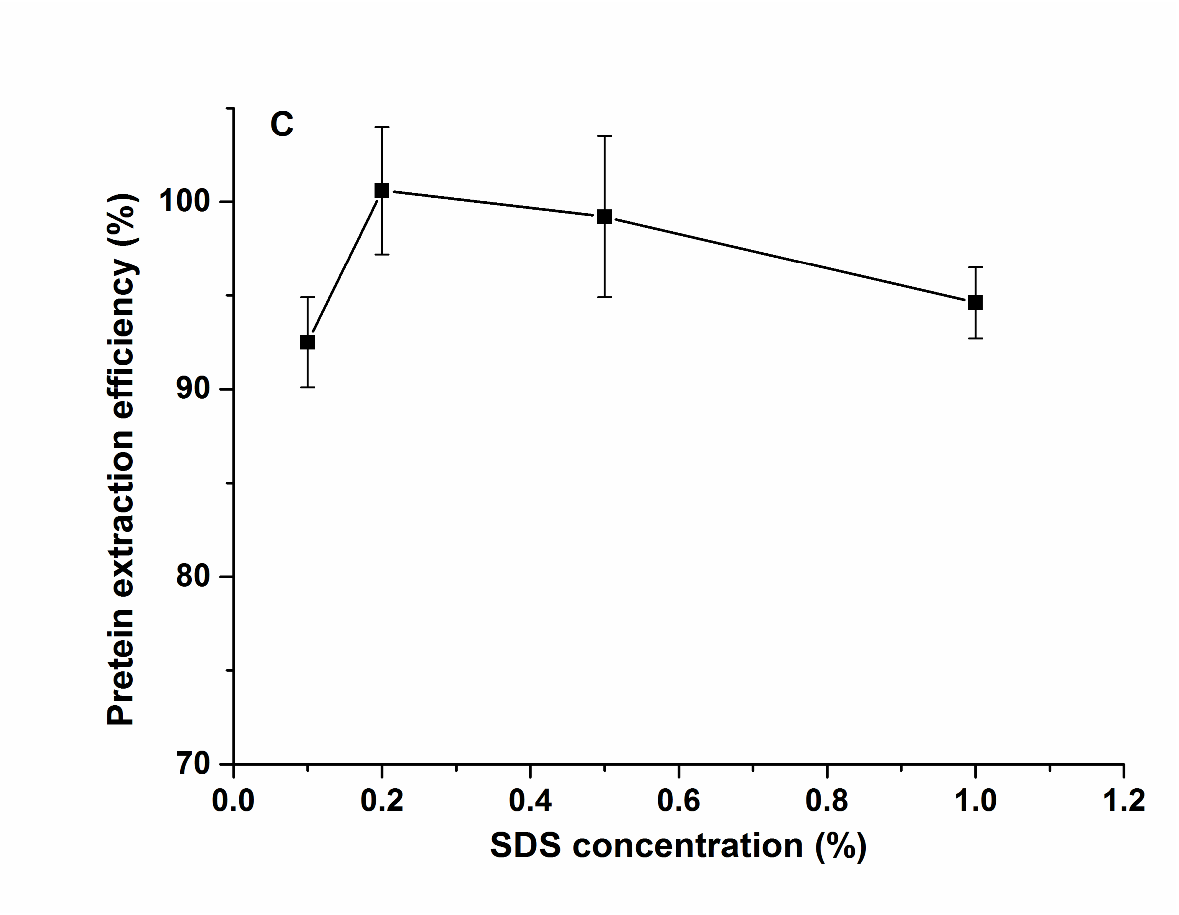

Supplemental Fig. 3 Optimization of sample extraction. salt concentration (A), concentration of urea (B) and SDS (C) on the protein extraction efficiency for the soybean seed.

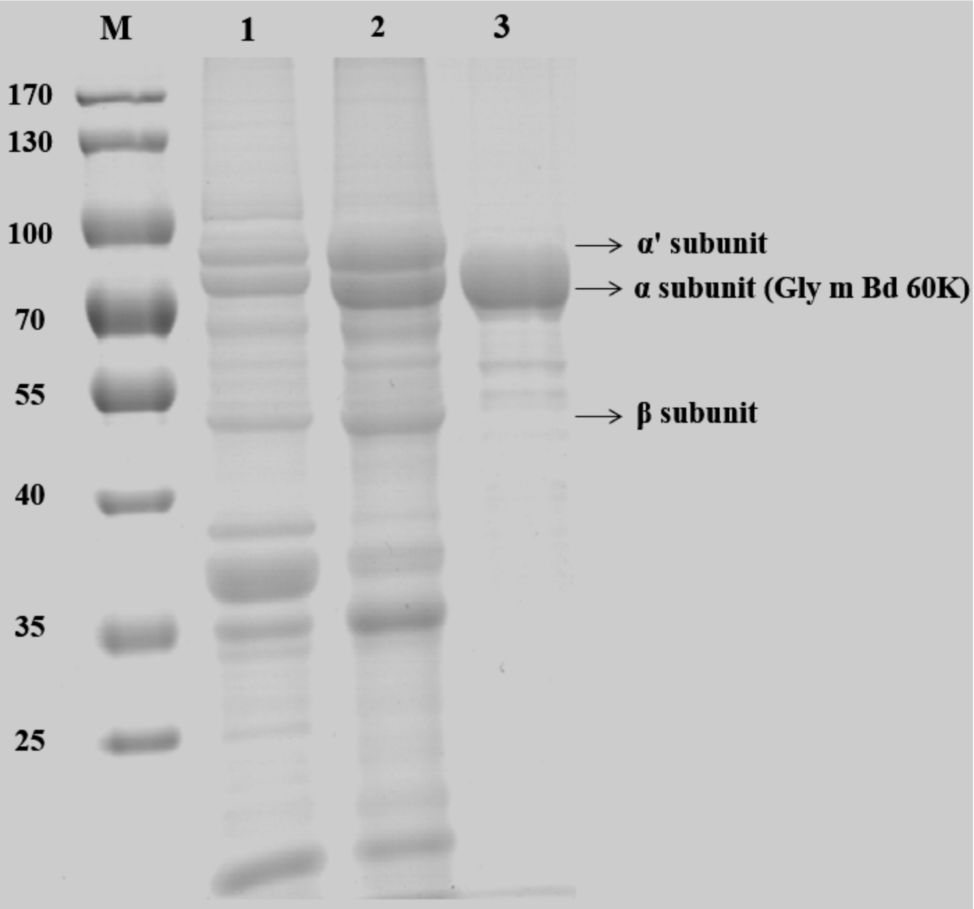

Supplemental Fig. 4 SDS-PAGE analysis of purified Gly m 5.0101. Lane 1, molecular weight standards; lane 2, soybean seed extract; lane 3, crude β-conglycinin extract; lane 4, Gly m 5.0101 purified by SDS-PAGE.

.

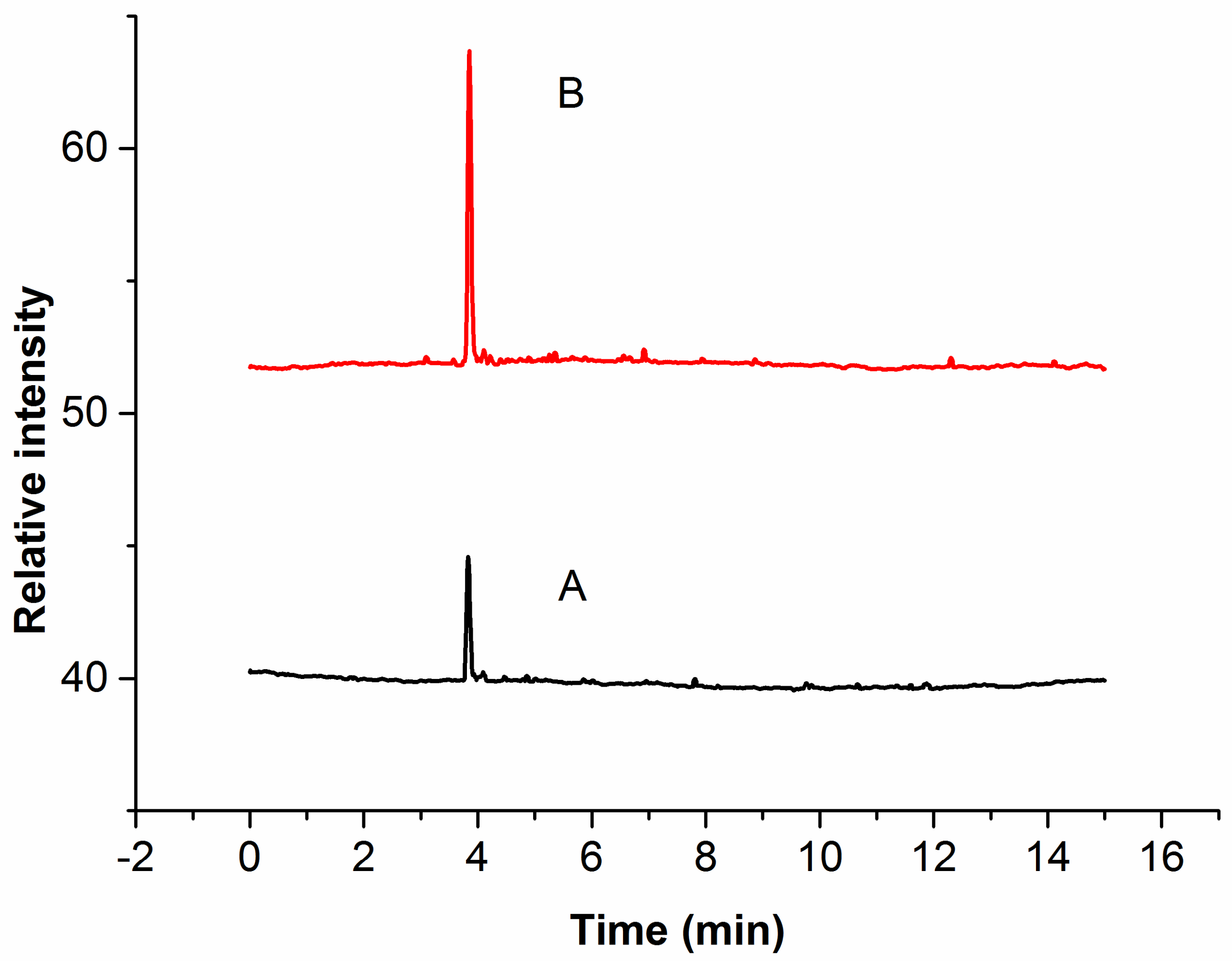

Supplemental Fig. 5. The limit of detection (A) and limit of quantitation (B) MRM chromatograms in soybean extract matix.

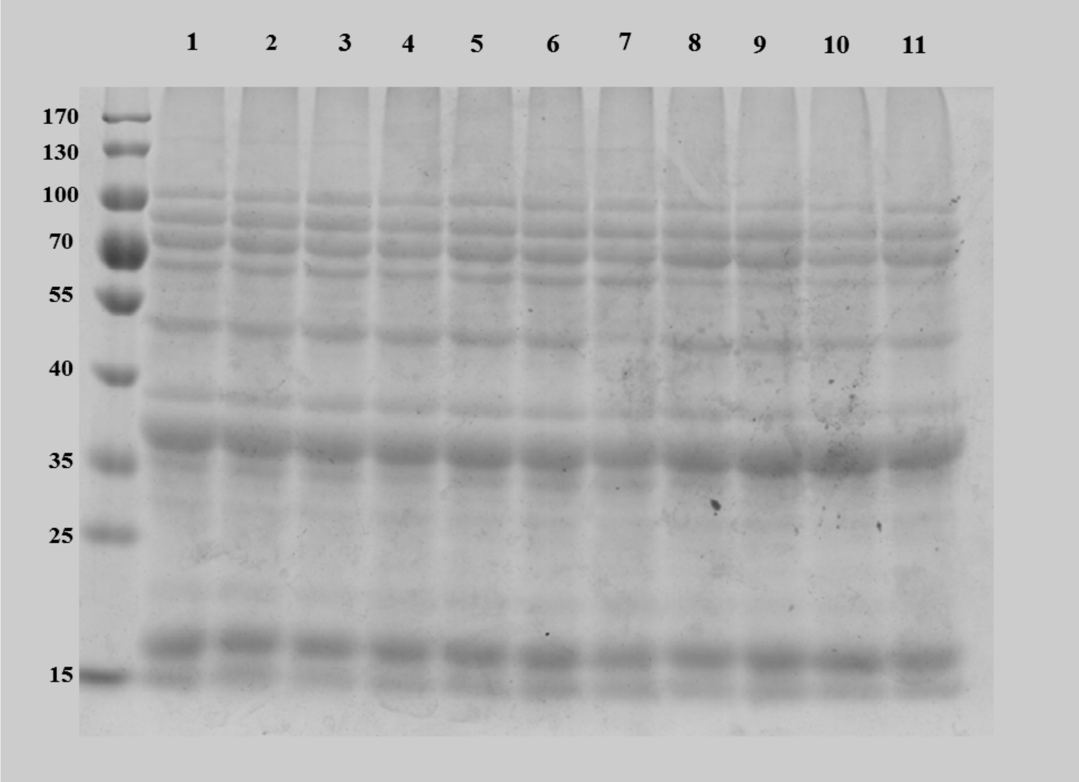
1 2 3 4 5 6 7 8 9 10 11
170
130
100
70
55
40
35
25
15

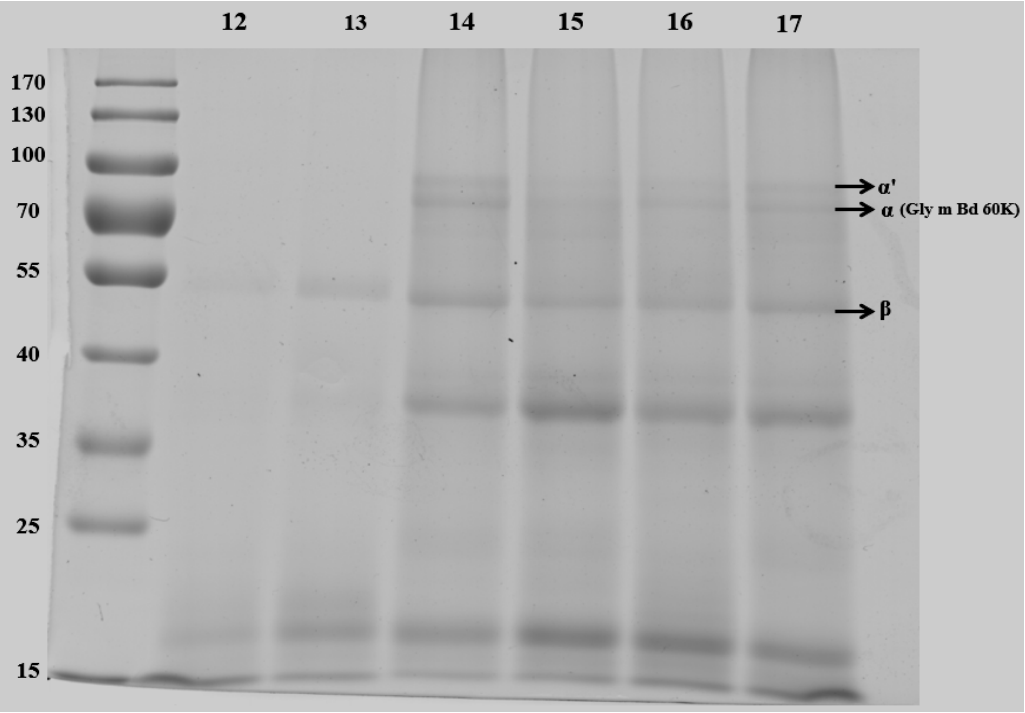
12 13 14 15 16 17
170
130
100
70
55
40
35
25
15
α'
α (Gly m Bd 60K)
β

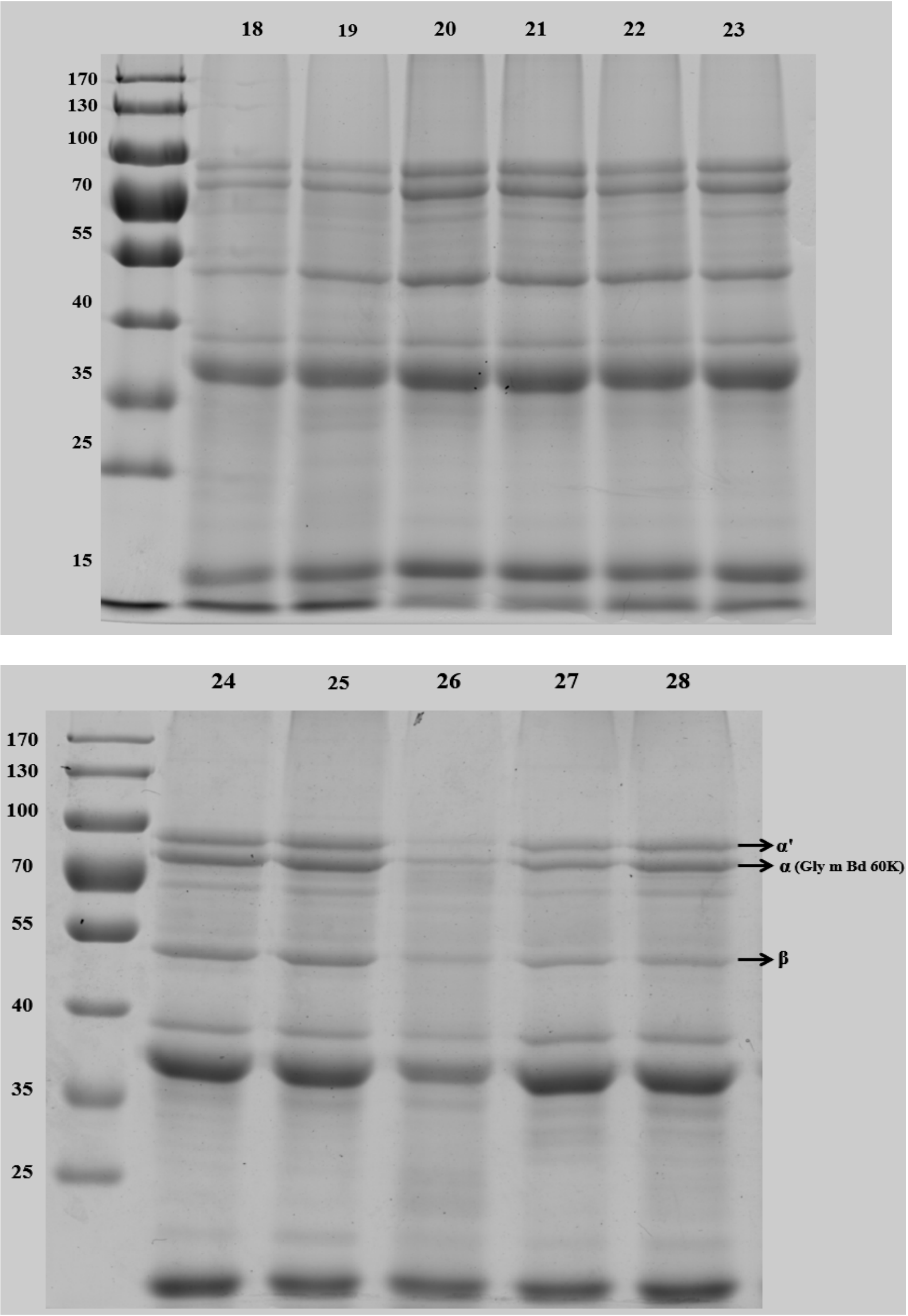

Supplemental Fig. 6 SDS-PAGE analysis of soybean seeds and soybean products. Lane 1-11, eleven soybean seeds; lane 12-17, six fermented soybean meals; lane 18-23, six extruded soybean meals; lane 24-28, five extruded full-fat soybeans. The standard molecular weight standards were shown on the left of each map.

Supplemental Table 1 Gradient elution procedure of NanoLC.

Time (min)	Flow rate (nL/min)	Mobile phase A (%)	Mobile phase B (%)
0	200	99	1
60	200	60	40
65	200	10	90
75	200	10	90
76	200	99	1
90	200	99	1
